# Simultaneous Absorbance and Fluorescence Measurements Using an Inlaid Microfluidic Approach

**DOI:** 10.3390/s21186250

**Published:** 2021-09-17

**Authors:** Joshua J. Creelman, Edward A. Luy, Gabryelle C. H. Beland, Colin Sonnichsen, Vincent J. Sieben

**Affiliations:** 1 Department of Electrical and Computer Engineering, Dalhousie University, 1360 Barrington Street, Halifax, NS B3H 4R2, Canada; js221530@dal.ca (J.J.C.); colin.sonnichsen@dartmouthocean.com (C.S.); 2 Dartmouth Ocean Technologies Inc., 25 Parker Street, Dartmouth, NS B2Y 4T5, Canada; Eddy.Luy@dartmouthocean.com; 3 Department of Process Engineering and Applied Science, Dalhousie University, 5410 Spring Garden Road, Halifax, NS B3J 1B6, Canada; gb995879@dal.ca

**Keywords:** lab-on-chip, microfluidic, PMMA, rhodamine, light absorbance, fluorescence, sensor

## Abstract

A novel microfluidic optical cell is presented that enables simultaneous measurement of both light absorbance and fluorescence on microlitre volumes of fluid. The chip design is based on an inlaid fabrication technique using clear and opaque poly(methyl methacrylate) or PMMA to create a 20.2 mm long optical cell. The inlaid approach allows fluid interrogation with minimal interference from external light over centimeter long path lengths. The performance of the optical cell is evaluated using a stable fluorescent dye: rhodamine B. Excellent linear relationships (R^2^ > 0.99) are found for both absorbance and fluorescence over a 0.1–10 µM concentration range. Furthermore, the molar attenuation spectrum is accurately measured over the range 460–550 nm. The approach presented here is applicable to numerous colorimetric- or fluorescence-based assays and presents an important step in the development of multipurpose lab-on-chip sensors.

## 1. Introduction

Lab-on-chip (LOC) sensors have emerged as a promising technology to reduce costs in environmental and diagnostic sampling [1]. By consolidating the chemistry and instrumentation from analytical laboratory techniques onto a network of microfluidic channels, expensive and time-consuming steps can be automated and streamlined. LOC further allows the collection of real-time data in remote settings by non-technical personnel. For example, current colorimetric LOC technologies have led to sensitive platforms for in situ measurement of nitrate/nitrite [2,3], phosphate [4,5,6], *E. coli* [7], and various heavy metals [8,9,10]. There are also LOC technologies which use electrochemical techniques, often used to monitor biological cell death and protein/DNA separation/purifications [11] and determining corrosion including corrosion caused by microbiology [12]. These field portable and in situ LOC devices are often sensing small volumes of fluid and as such typically use electrochemical detection or optical spectroscopy interrogation. Absorption and fluorescence detection are the mainstay approaches to optical spectroscopy due to their simplicity [13]. While LOC platforms have enabled widespread and low-cost sensing, it would be advantageous for microfluidic devices to perform combined spectroscopic measurements on the same sample under investigation.

Coupling light to and from the microfluidic devices is readily attained on the laboratory bench. Even without a microfluidic channel, waveguiding approaches have been used to couple light to and from fluid measurement cells. One example of using a waveguide was in detecting rhodamine 6G fluorescence shifts within porous glass (PG). A waveguide-core was physically integrated with the PG structure implanted with rhodamine. The material–dye complex was excited and the emitted fluorescence obtained in the presence of ethanol [14]. Another example is the measurement of refractive index changes using waveguide coupling with Corning Gorilla Glass, commonly used in smartphones. Adding waveguiding to these screens was purported to increase the functionality of the screen [15]. When the measurement cell is contained within a microfluidic chip, fibre optic cables are often inserted as waveguides to avoid manufacturing complexity in realizing hybrid-material devices for attaining the refractive indices for waveguiding [16,17]. Others have separated the optical waveguiding chip from the fluid handling chip; for instance, Okubo et al. used silicon nitride waveguide chips that have been integrated with microfluidic handling chips to measure biotin/streptavidin with LODs of 130 nM [18]. Integrating microfluidic handling with optical components, like waveguides, to perform on-chip spectroscopy can enable higher degrees of miniaturization [19,20].

There have been several reports of microfluidic sensors with the ability to simultaneously detect fluorescence and absorbance signals [21,22,23]. Recent advances have demonstrated the ability to distinguish multiple analytes through simultaneous absorption and fluorescence [21] or couple scattering, fluorescence and absorption into a single device [22]. Simultaneous fluorescence and absorbance microfluidic measurements of droplets have also been shown to be able to detect pH gradients of 3.9–8 in real-time with a fluorescence detection limit of 400 nM fluorescein in water [23]. Yang et al. accomplished this by using a laser coupled to a microfluidic chip via waveguides for measuring picolitre volumes in a 10 mm long optical cell; the fluorescence was measured from the side of the same optical cell, with a cross-section of 35 µm × 26 µm [23]. Fibre optics and waveguides have been a robust tool for guiding light to interrogate fluids since the early 1990s [24]. However due to the nature of in situ instruments, shock and vibration can lead to physical misalignment that is often addressed by using adhesives and epoxy bonding of the fibre to the waveguide/carrier. The main challenge with adhesives arises if the chip must be replaced or serviced, and when packaging the overall instrument to fit into a submersible canister, where the minimum bend radius must be respected. Another important difficulty for realizing simultaneous optical measurements lies in creating a long-pathlength absorption cell for low analyte concentrations. For sensitive measurements, effective path lengths of 10–100 mm are typical for absorbance cells, such as the work of Floquet et al. [25]. On bench, Floquet et al. demonstrated absorbance cell path lengths that ranged from 25–100 mm for nanomolar detection of iron and manganese, and Milani et al. deployed these chips in natural waters based on a 100 mm flow cell [26]. Additionally, these pathlengths allow for nanomolar concentrations of key limiting nutrients nitrate and phosphate [3,27,28]. Cavity enhancement is a proven solution to decrease physical path lengths down to 50 µm with reflective surfaces while maintaining absorbance measurements in the micromolar range [29]. Through cavity-enhanced absorption spectroscopy (CEAS), the light from the source bounces back-and-forth through the droplet sample to enhance the path length by a factor of 28 [29], which yielded an effective pathlength of 1.4 mm. Rushworth et al. used thymol blue indicator dye in a range of 50–2000 µM, measured using CEAS with a 1s integration time [29]. The authors noted that reliably placing and aligning mirrors in such a microfluidic cell for CEAS-based approaches can be challenging. Optical fibres are another solution for increased sensitivity in absorption experiments, through improved coupling of light into and out of the sample area [13]. However optical fibres may introduce complexities when mated with a microfluidic channel, often relying on UV-curable or time-set epoxies that can hinder scaled manufacturability, as well as having minimum bend radii that can restrict miniaturization efforts. It is, therefore, ideal to have more optical elements integrated on-chip when robustness and field portability are eventual requirements.

Measuring both fluorescence and absorbance simultaneously covers a wide range of potential applications. For example, the Cyclops-7F commercial fluorometer from Turner can measure various target substances like chromophoric dissolved organic matter (CDOM), protein pigments in algae, fluorescent dyes, oils, etc. Fluorometers simply change the filter set and excitation source to match the target substance’s absorbance and fluorescence spectra. Fluorometers are also capable of measuring substances lacking native fluorescence, like real-time PCR (RT-PCR) devices, provided reagents and automated protocols are integrated with the optical technique. Such devices use fluorophores which bind to DNA and therefore fluorescence can be used to quantify the DNA. The work of Preston et al. demonstrates the utility of microfluidics on an in situ quantitative PCR (qPCR) platform [30]; where, portable or in situ versions of these devices aim to minimize reagent consumption and sample waste. Fluorescence is also used for taxonomic discrimination of phytoplankton [31]. The work of MacIntyre et al. involves detection of harmful algal bloom (HAB) forming organisms, both quantifying them [32] and differentiating between viable and unviable cells [33]. However, it is notable that fluorescent pigment protein content can vary cell-to-cell and species-to-species [34], so it is advantageous to have a multiparameter analysis when evaluating environmental samples. Dissolved organic matter (DOM) also plays a role in HAB formation [35]. There are several studies of CDOM which require both absorbance and fluorescence measurements, specifically when determining seasonal changes in the optical properties [36,37,38]. Algae fluorescent measurements are influenced by CDOM as they can share an overlap in fluorescence spectra [39]. Therefore, simultaneous detection of fluorescence and absorbance better contextualizes the sample under investigation than a single measurement, producing more accurate differentiation of chromophores and fluorophores than either measurement alone.

### Novel Flow Cell Design

We have developed a microfluidic optical system capable of simultaneous detection of absorption and fluorescence signals in liquid samples, without physically attaching optic components to the chip. The novel cell presented here is based on an approach we used for absorption spectroscopy [27], where clear and opaque poly(methyl methacrylate) (PMMA) is used to define optical paths and selectively block background light. By spatially separating excitation and fluorescence optical paths and using absorptive filters, backgrounds and scattered signals are minimized in our fluorescence detection. This microfluidic cell is characterized using a rhodamine B dye, and we find high sensitivity and low detection limits in both absorbance and fluorescence detection.

**Figure 1:**
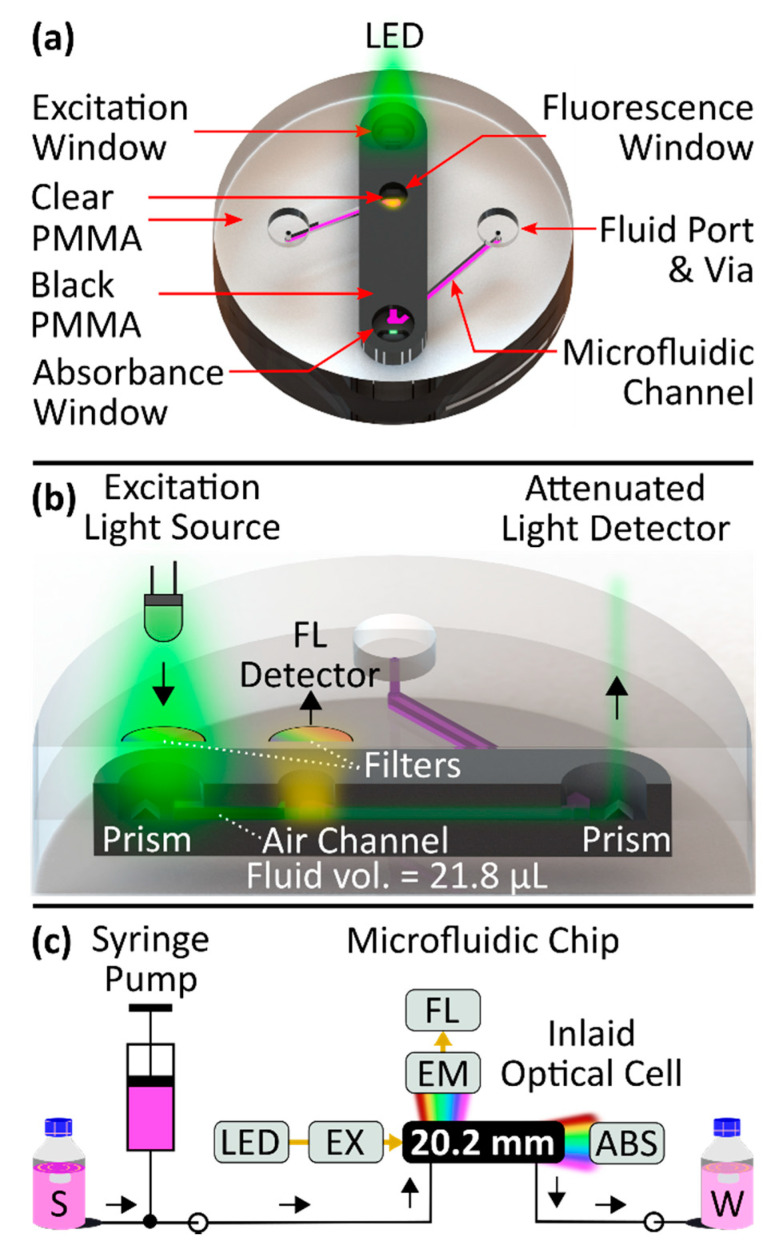
(**a**) Top perspective view of the microfluidic device with labelled features. The three windows labelled Excitation, Fluorescence and Absorbance are sections of clear PMMA interwoven within the opaque PMMA to create optical viewing ports. The fluid ports and vias are used to connect tubing to the optical cell. (**b**) Novel inlaid optical cell based on a microfluidic channel with off-chip optical components. Black PMMA is embedded within clear PMMA to block extraneous light and create integral windows for excitation and for observing fluorescence and absorbance of the sample. The optical path comprises a total sample volume of 21.8 µL. The LED illumination is shown as a triangle of green light incident upon the prism but is a 3D-cone with a non-uniform intensity distribution. (**c**) Characterization apparatus for validation of the microfluidic cell using the fluorescent dye rhodamine B. Optical measurements are obtained using two spectrophotometers for absorbance (ABS) and fluorescence (FL). Excitation (EX) and emission filters (EM) are implemented to prevent overlapping of LED and fluorescence spectra.

Figure 1a shows a top-down view of our novel microfluidic optical cell configuration. In this design there are three optical windows down the centre of the chip, each with the purpose of allowing light to bypass the opaque inlay. The first window—the excitation window—transmits light into the microfluidic chip from the LED source. The second window—the fluorescence window—is used to measure fluorescence of a contained fluid sample. The third window—the absorbance window—is used to measure the absorbance of that same fluid sample over a 20.2 mm path length. The location of each window is labelled in Figure 1a, along with the fluid ports which allow sample to pass through the system.

A cross-sectional view of the chip can be seen in Figure 1b. The chip design uses embedded microprisms, inspired by the work of Grumann et al. [40], to couple light through total internal reflection into the optical channel to excite the sample. Two of the optical windows have light filters mounted above them; the excitation window has an excitation filter mounted above it and the fluorescence window has an emission filter mounted above it. A portion of the filtered emitted rays pass through the excitation window and reflect off an embedded microprism towards an air-filled rectangular channel. The air-filled channel defines an optical path through the opaque PMMA. A channel bridging the prism and the fluorescence chamber window is necessary to transmit the excitation light, while permitting physical space for the optical filters that sit above the microfluidic chip. The fluorescence chamber could be further along the absorbance channel; however, the fluid properties would uncontrollably interfere with the emission given that the absorbance would attenuate the signal before it reaches the fluorescence well. After light passes through the air-filled channel, shown in Figure 1b, it enters the fluorescence well, located within the fluorescence window (clear PMMA), stimulating fluorescence. The fluorescent emissions then exit the fluorescence window and are filtered by the emission filter before reaching the fluorescence detector. The light originating from the source continues down the long microfluidic channel (opaque PMMA) to the absorbance window. At the absorbance window (clear PMMA) a light detector is mounted to measure the attenuated light, directed by a second microprism located within this window.

Figure 1c shows a flow diagram detailing the automated system used to characterize our optical cell. Ultimately, we used this setup to demonstrate nanomolar limits of detection are possible for both absorbance and fluorescence using our novel microfluidic optical cell.

## 2. Materials and Methods

### 2.1. Chip Fabrication

**Figure 2:**
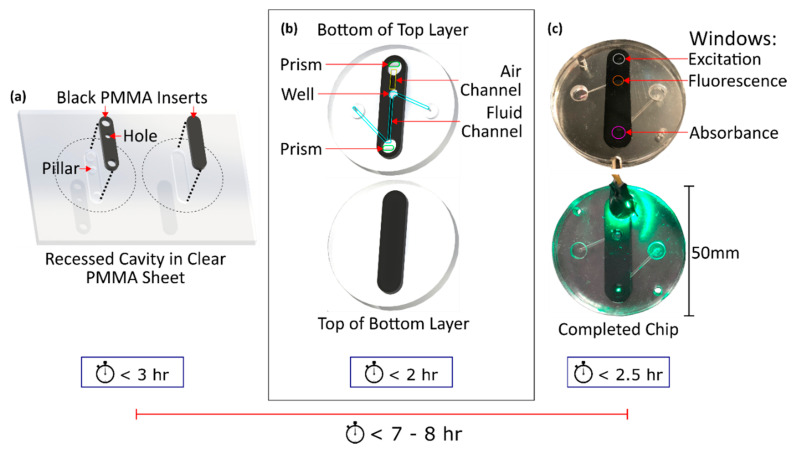
Fabrication process overview for creating the inlaid microfluidic absorbance & fluorescence chip. The process is broken into 3 steps that collectively take 7–8 h. (**a**) Step 1 involves inlaying black PMMA inserts into recesses in a clear PMMA sheet. (**b**) In step 2, microchannels, prisms, and wells are milled into the inlaid sheet. The milled microchannels cross the clear-black material interface to realize integral optical windows in an opaque cell for both absorbance and fluorescence observations. Individual chip halves are cut from the sheet to prepare for bonding. (**c**) In step 3, the two halves or layers are exposed to a solvent vapour and then bonded together under temperature and pressure to yield the devices shown. Top is a photograph of the final chips without excitation, and the bottom is a photograph with an LED directed to the excitation prism, where the absorbance prism shows light that only propagated through the microfluidic channel.

A bilayer microfluidic chip with an inlaid optical cell was used to measure absorbance and fluorescence. The process of inlaying opaque PMMA into clear PMMA has been described at great length elsewhere [27] and will be summarized here. First, a top and bottom black insert were cut from a sheet of black extruded PMMA (9M001, Acrylite, Sanford, ME, USA) using an Epilog Mini24 (Epilog, Golden, CO, USA). The top and bottom inserts were designed for inlaying into the top and bottom layers of the chip, respectively. In Figure 2a, the design of both inserts is clearly visible. Each insert is comprised of a straight thin section of black PMMA with rounded ends. Three cylindrical cuts were made in the top black insert: upon completion, these would act as optical windows into/out of the optical cell. The two cuts at either ends had diameters of 6 mm while the middle cut had a 4 mm diameter. The bottom insert was identical to the top but without the described cylindrical cuts. Interweaving clear and black PMMA in this manner permits integral optical windows, where the microfluidic channel crosses the hybrid-material boundary.

Matching cavities were then machined from a sheet of clear extruded PMMA (0A000, Acrylite, Sanford, ME, USA) for each insert created by a LPKF S103 micro-mill (LPKF, Garbsen, Germany). These cavities are shown in Figure 2a. The dimensions of each cavity matched their corresponding insert with a circumferentially added 25 µm, necessary to ensure that the inserts would press into the cavities with uniformity. Pillars were left in the clear PMMA which would correspond to the holes made in the black PMMA inserts, such that when the black PMMA was inserted a hybrid black–clear substrate was created. After a dry fit, both inserts and cavities were then exposed to chloroform, hand-pressed into their cavities, and temperature and pressured annealed to realize a seamless bond, important for microfluidic channel routing. The bonding process was modified from the original work of Ogilvie et al. [41], the pressured used was 625 N cm^−2^ at a temperature of 115 °C for 2.5 h.

After inlaying black PMMA into the sheet of clear PMMA, the entire sheet was backplaned by 200 µm for creating a uniform thickness and then features were milled. The added features included: microchannels, prisms, vias, and syringe ports, as shown in Figure 2b. Two different channels were created. The first, the fluid channel, had a width and depth of 1 mm, but smaller channels down to 400 µm are readily possible as we have demonstrated [27]. The fluid channel was cut with a “z-shaped” geometry as seen in the top part of Figure 2b highlighted in light blue. Z-shaped channels are typical for light absorbance measurements [27,28]. However, to increase the amount of fluorescence measured, a fluorescence “well” was added to the channel. This well is labelled in Figure 2b as a rounded section at the first end of the fluid channel with a larger diameter. The well increased the volume of fluid interrogated through the fluorescence measurement window, thereby increasing the fluorescence signal measured by the detector. The fluid channel could be filled with fluid by screwing a syringe or tubing into either syringe port. The second channel, an air channel, was 2 mm wide, 1 mm deep, and 7.7 mm long. This channel provided a path for light reflecting off the incident prism to reach the fluid channel. Only a portion of the reflected light rays traversed the air channel to reach the fluid channel, and ultimately the detector. Rays beyond the solid angle to the detector area are blocked by the opaque PMMA.

The completed top and bottom discs of the microfluidic chip were then cut from the PMMA sheet. The discs were bonded together using a thermal, solvent, and pressure process to seal the channels, with identical parameters used for the insert bonding. The pressing temperature, however, was lowered to 85 °C to prevent channel distortion. After pressing, assembly of the microfluidic chip was complete. A photograph of the completed chip is shown in Figure 2c, where an LED is shone on the incident prism, light is passed through the fluorescence well, and finally reaches the absorbance window/prism visible by the green dot at the bottom centre of the chip. The complete fabrication process of this chip took approximately 7–8 h. There were no coatings applied the PMMA during or after production of the chip.

### 2.2. Chemistry

The inlaid cell was evaluated through bench tests using calibration standards. Rhodamine dyes are excellent calibration standards due to their photostability and high quantum yield [42]. The optical performance of the chip was calibrated using seven different concentrations of the fluorescent dye rhodamine B. Milli-Q water was used as the solvent. The concentrations of each sample were: 0.1, 0.25, 0.5, 1, 2, 5, and 10 µM. First, a 2.5 g/L stock solution of rhodamine B was prepared by diluting 0.5 g of rhodamine B (C_28_H_31_ClN_2_O_3_, ≥95%, R6626, CAS 81-88-9, Lot # SHBL5990, Sigma-Aldrich, Oakville, Ontario, Canada) with Milli-Q water to a total volume of 200 mL. The various standards were then prepared through serial dilution of the 2.5 g/L (5219 µM) stock.

### 2.3. Experimental Setup

A syringe pump is used to draw from a fluid reservoir—S in Figure 1c—using tubing. The output of the syringe pump is connected to the input of the microfluidic chip. The fluid travels through the inlaid optical cell for inspection by the excitation light source. Measurements of light absorbance and fluorescence are captured simultaneously by two spectrometers. The total amount of light absorbed by the fluid in the 20.2 mm long optical path is measured using the first spectrometer (USB2000+, Ocean Optics, Orlando, FL, USA). Similarly, a portion of the light emitted by the fluid through fluorescence is measured simultaneously using the second spectrometer (Flame, Ocean Optics). After the measurement time window, the fluid exits the chip and travels via tubing to waste—W in Figure 1c.

Injection of fluids through the microfluidic chip was achieved using several off-the-shelf components. A Vici Cheminert C65Z 10-port selector valve (Valco Instruments Co. Inc., Houston, TX, USA) was used to pull from different samples without cross-contamination. FEP tubing connected each sample to a respective intake port on the valve. The output connected to the intake of an off-the-shelf Cavro XC syringe pump (Tecan Systems, San Jose, CA, USA) which was used to drive fluid flow. The output of the syringe pump was directly connected to the input of the microfluidic chip. Fluids were pumped through the chip at a consistent flow rate of 1.5 mL/min, and flow was paused during each sample/blank measurement.

Rhodamine B excitation was achieved using an LED centred at λ = 521 nm (Cree C503B-GANCB0F0791-ND, measured FWHM ≈ 35 nm). Excitation and emission filters were implemented—mounted on top of the optical windows—to prevent overlapping of spectra. A 550 nm short-pass filter (Edmund Optics, 84–695) was used as the excitation filter and was mounted onto the chip between the LED and the excitation optical window. A 578 nm bandpass filter with a 16 nm bandwidth (Edmund Optics, 87–738) was used as the emission filter—mounted between the fluorescence window and the spectrometer. Both filters are of 6 optical density and so the wavelengths from the LED overlapping that of the fluorescence readings are suppressed. Although no additional optics were used, plano-convex and or GRINS lens could be used to collimate and focus light at each window to improve light collection efficiency.

Both spectrometers were connected to a personal computer via USB. Data acquisition was controlled using software supplied by the manufacturer. A 20 ms integration time was used for the absorbance measurements and a 10 s integration time for the fluorescence measurements. The reason for such a difference in integration time is due to the fluorescence emissions being much weaker in comparison to attenuated light in the samples we were observing. Three absorbance and three fluorescence scans were averaged together to minimize measurement noise. All optical components were aligned and mounted to the chip in a 3D printed optics holder.

### 2.4. Analytical

A measurement of the background light was acquired by each spectrometer at the beginning of testing by powering off the LED light source. The background light was minimized during testing by placing a black box enclosure around the entire setup and detectors. The dark reference was subtracted from each capture by the data acquisition software to account for noise in the electronics acquisition. Before each sample measurement, a blank measurement was captured by filling the optical cell with Milli-Q. Blank measurements help quantify any carryover between successive sample measurements and provide a reference spectrum from which absorbance and fluorescence can be calculated. At each wavelength, λ, absorbance is calculated according to the Beer–Lambert law:

(1) $$A\left(\lambda \right)=-\log_{10}\left(\frac{S_{a}\left(\lambda \right)}{B_{a}\left(\lambda \right)}\right),$$

where A(λ) is absorbance. $S_{a}\left(\lambda \right)$ and $B_{a}\left(\lambda \right)$ are the counts of the spectrometer at the absorbance window when sample and blank are in the optical cell, respectively. Fluorescence at each wavelength is calculated as a differential of sample and blank:

(2) $$F\left(\lambda \right)=S_{f}\left(\lambda \right)-B_{f}\left(\lambda \right),$$

where F(λ) is fluorescence. $S_{f}\left(\lambda \right)$ and $B_{f}\left(\lambda \right)$ are the counts of the spectrometer at the fluorescence window when sample and blank are in the optical cell, respectively.

The absorbance and fluorescence of each sample concentration is measured in triplicate to assess measurement repeatability. The following details the measurement procedure of any given concentration. First, 3 mL of Milli-Q is pumped into the chip. Under static flow conditions, the readings of both spectrometers are recorded simultaneously following the parameters described in Section 2.3. Next, 3 mL of sample is pumped into the chip, flow is stopped, and the new readings are recorded. This is performed three consecutive times to acquire three independent absorbance and fluorescence measurements of each concentration.

## 3. Results and Discussion

### 3.1. Absorbance and Fluorescence Spectra

**Figure 3:**
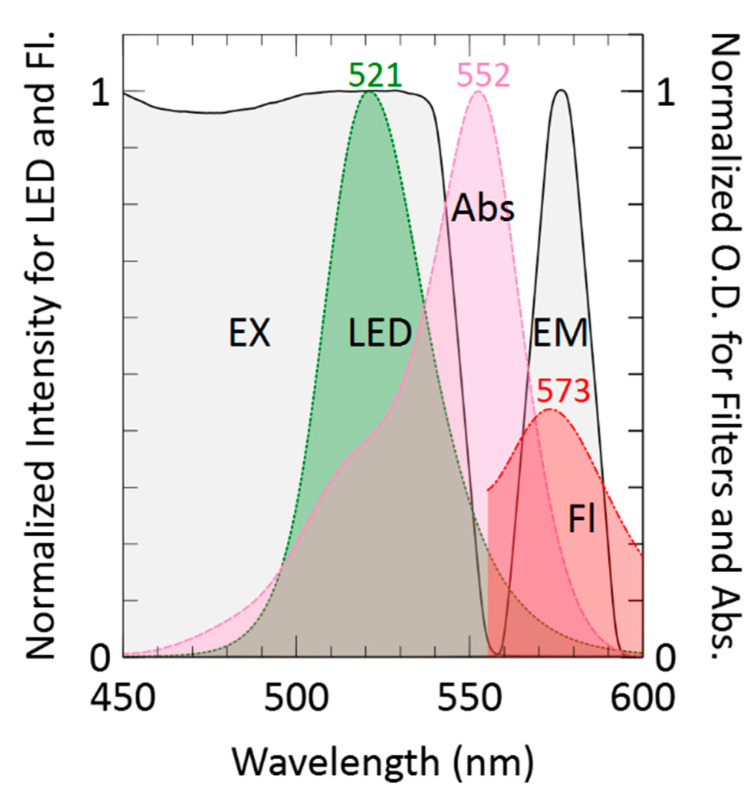
Measured LED emission (green) and rhodamine B absorbance (pink) spectra normalized to their maximum values. Also shown is a measured rhodamine B fluorescence spectrum (red), where the amplitude of the fluorescence curve peak is arbitrary scaled for visualization. Finally, the measured transmission spectra of the excitation (EX) and emission (EM) filters are shown in black. Axis Y1 shows normalized intensities for LED emission and fluorescence plots. Axis Y2 shows normalized O.D.s for ex/em filters and absorbance.

We first characterized the optical properties of the system using a white-light LED (RL5-W18015, superbrightleds.com). The measured absorbance spectra of a rhodamine B sample are shown in Figure 3. The pink dashed curve shows an absorption peak at λ = 552 nm. Likewise, the measured emission spectrum of the excitation LED is shown in Figure 3 as a green dotted curve. The transmission spectra of both the excitation and emission filters are shown as black curves with areas shaded in transparent-grey. The fluorescence spectra can be seen in Figure 3 as a red dashed–dotted curve showing an emission peak at λ = 573 nm. To acquire the fluorescence spectrum, a 15 µM rhodamine B sample was excited with the green LED and the output spectrum was captured without filters. As such, the rhodamine B fluorescence emission spectrum was determined by subtracting the green LED spectrum scaled to the LED’s peak at 521 nm. In summary, Figure 3 shows that given the rhodamine B fluorescence spectrum (arbitrarily scaled for visualization), the LED and the excitation–emission filter set are selected for minimizing spectral overlap.

### 3.2. Calibration

**Figure 4:**
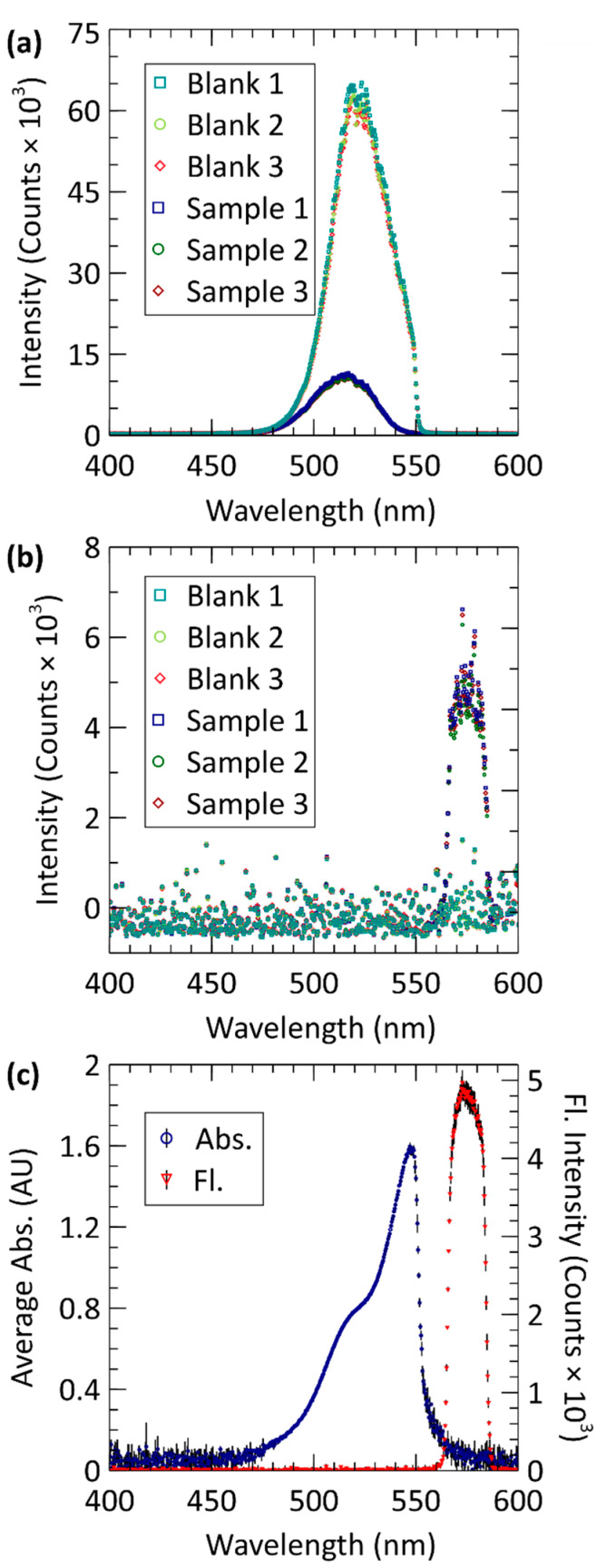
Simultaneous absorbance and fluorescence data acquired from the microfluidic inlaid cell for a 10 μM rhodamine B. (**a**) Intensity measured from the absorbance optical prism and spectrometer. (**b**) Intensity measured from the fluorescence optical window and spectrometer. For both (**a**) and (**b**), blank and sample spectra are plotted in triplicate. (**c**) Calculation of absorbance from the averaged intensity data in (**a**) using the Beer–Lambert Law, as well as the fluorescence intensity data that is the average of the three measurements. Black vertical lines are one standard deviation.

To evaluate the performance of our novel microfluidic spectroscopy cell, we started calibration with a 10 μM sample of rhodamine B. The sample was measured in triplicate using the methods and parameters described in Section 2. Figure 4 shows the data obtained from the three blank and three sample measurements. The order of measurement was blank 1, sample 1, blank 2, sample 2, blank 3, and sample 3 to rule-out crosstalk between samples. Figure 4a plots the measured counts of the absorbance spectrometer for each blank and sample measurement. Lighter colours of blue, green, and red depict data from blanks 1–3. Similarly, darker hues of blue, green, and red depict data from samples 1–3. As seen in Figure 4a, blank and sample absorbance measurements were very consistent with blank intensities ranging from 62–65 k counts with a 1.27 k standard deviation and sample intensities ranging from 7.8–8.2 k counts with a standard deviation of 238 within a 5 nm window centred at 527 nm. Likewise, Figure 4b uses the same colour scheme to plot the measured counts of the fluorescence spectrometer for each blank and sample at 10 µM, which can be seen to have good consistency. The counts in the wavelength range of 570–575 nm were averaged for triplicate blanks and standards. The average blank data points range from −20–12 with a standard deviation of 16 and the average sample data ranges from 4.7–4.9 k counts with a standard deviation of 140. The signal-to-noise ratio was higher in the fluorescence measurements than the absorbance measurements due to fluorescence being a much weaker signal and requiring a significantly higher integration time, 50 times that of absorbance. Nevertheless, the measured data are still highly consistent.

Figure 4c plots the calculated absorbance and fluorescence versus wavelength using the data from Figure 4a,b. The absorbance of the 10 μM rhodamine B sample, averaged from each of the three measurements, is plotted against wavelength and shown as blue circles. Similarly, the averaged fluorescence is plotted against wavelength and shown as red upside-down triangles. In both cases, vertical black error bars show the standard deviation associated with each data point, calculated from the three measurements at each wavelength. The average standard deviation associated with absorbance is 0.012 AU between 470–570 nm and with the fluorescence curve is 60.7 counts from 550–600 nm. This region encompasses the transmission spectrum of the light source to the emission spectrum of the sample and contains all relevant spectral data. Beyond this region, measurements are near-zero and consist mainly of noise. The small error bars, shown by both curves indicates a high level of measurement consistency.

**Figure 5:**
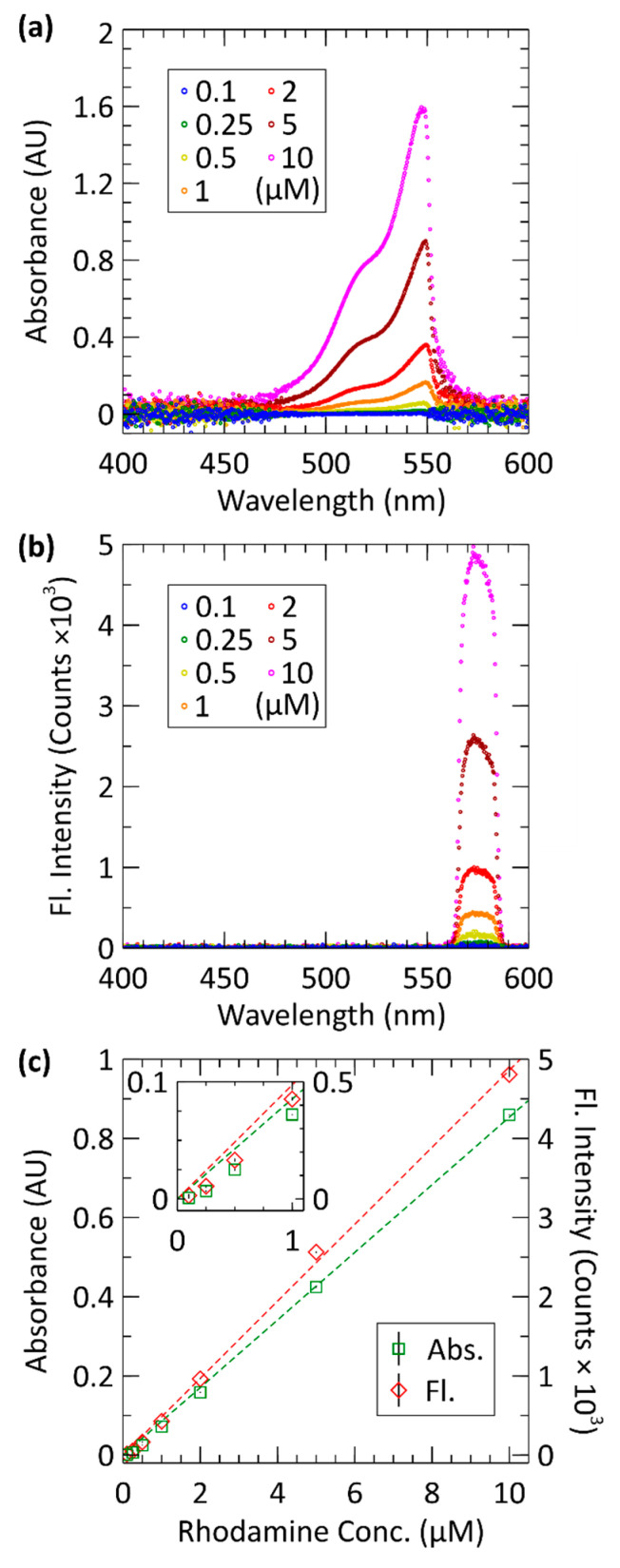
Rhodamine B calibration data for samples from 0.1 to 10 µM; measured in triplicate. (**a**) Averaged absorbance vs. wavelength for each sample. (**b**) Averaged fluorescence vs. wavelength for each sample. (**c**) Absorbance at 525–530 nm and fluorescence at 570–575 nm plotted against rhodamine B concentration. Inset shows the measured data at low concentrations from 0.1 to 1 µM.

Upon successful proof-of-concept testing with the single 10 μM rhodamine B sample, a full rhodamine B calibration was performed. Figure 5 shows the results of a rhodamine B calibration series. Seven samples of rhodamine B ranging from 0.1–10 µM were analysed following the procedure detailed in Section 2.4. Figure 5a plots absorbance vs. wavelength for a series of rhodamine B samples. Each curve, differentiated by colour, represents a different sample concentration—labelled in the legend of the plot. Each concentration was measured in triplicate: thus, each curve represents the averaged absorbance vs. wavelength data. The expected Beer–Lambert trend can be observed in the data such that more concentrated samples of rhodamine B absorbed more of the incident light source. The sharp drop in the absorbance data near λ ≈ 550 nm is the result of the cut-off of the excitation optical filter. Figure 5b plots fluorescence vs. wavelength for the same samples, acquired at the same time as the absorbance measurements of Figure 5a. Sample concentrations are differentiated by colour using the same colour scheme as Figure 5a. The measured fluorescence of each sample had a sharp cut-off on either end due to the cut-off of the emission filter mounted to the fluorescence optical window. As seen in the plot, the lowest concentration sample produced the lowest amount of fluorescence—shown by the blue data points. As sample concentration increased, so too did their emitted fluorescence output. The most concentrated sample, 10 µM, produced the most fluorescence as expected by this trend—shown by the pink data points.

Figure 5c shows the processed data from Figure 5a,b. Here, both absorbance and fluorescence are plotted against concentration. Absorbance measurements are in green while fluorescence measurements are in red. The absorbance of each sample concentration is calculated as the average absorbance over a 5 nm window centred at 527 nm. Likewise, the fluorescence of each is calculated by averaging the fluorescence measurements between 570–575 nm. A line of best fit between absorbance and concentration was calculated such that A = (0.0854 ± 0.0002)C. Using A = 0.08535C a root-mean-squared-error (RMSE) of 0.0126 and R^2^ value of 0.998 was found. Similarly, a line of best-fit between fluorescence and concentration was calculated such that F = (486 ± 11)C. Using F = 486C a RMSE of 77.5 and R^2^ value of 0.998 was found. This demonstrates that, in either case, a linear model accurately describes the data.

### 3.3. Detection Limits

The limit of detection (LOD) can be defined as three times the standard deviation using the triple-sigma method [27,28,43,44]. The LOD for absorbance was calculated using the blanks preceding the 0.1 µM measurements, which ensured minimum crosstalk for evaluating the LOD. First each blank measurement was averaged over a 5 nm window centred at 527 nm as this is what was used when determining absorbance for the sample measurements. The data in the blank measurements ranged from 48 k–50 k counts, with a standard deviation of 666 counts. This results in 0.006 AU against the average blank; therefore, the triple sigma method yields 0.018 AU and when using the line of best fit from our calibration curve, the absorbance LOD equates to 208 nM rhodamine B. The LOD for fluorescence was similarly calculated using the same blanks but measured from the fluorescence window. The intensities were averaged between 570–575 nm. The data in the blank measurements ranged from 24–40 intensity counts, with a standard deviation of 7.6 counts, therefore the triple-sigma method yielded 23 counts of intensity. Using the line of best fit from our calibration curve, the fluorescence LOD equates to 47 nM rhodamine B The LOD is satisfactory for our intended deployments relating to dye tracer experiments, algae pigment measurements, and determination of hydrocarbon content in marine waters at nanomolar levels; however, there is room for improvement. For a point of reference, the Turner Cyclops-7F is a commercially available marine fluorometer and states a 0.01 ppb rhodamine or 0.02 nM is the LOD. However, this sensor is not a microfluidic device and the volume of interrogation from the LED beam into the water is orders of magnitude higher than the 20 µL of volume contained in our fluorescence chamber. Our limit of detection of 47 nM is significantly higher than such instruments. To achieve a better LOD the use of collimating optics, like GRIN lenses, and signal processing such as lock-in amplification can be implemented and would considerably improve the LOD. For example, the microfluidic chip for measuring fluorescence of rhodamine 6G by Pais et al. that showed an LOD of 100 nM [45] was improved to 10 nM using a lock-in amplification approach by Banerjee et al. [46]. We are investigating both optical and signal processing techniques to improve the LOD for future work, but the current LOD is in line with other microfluidic devices and is suitable for a wide range of experiments in situ [47]. Until these improvements are made, perhaps a more practical metric for the lower limit of the measurement range of our optical cell is the limit of quantification (LOQ), defined as ten times the blank baseline noise. Therefore, the inlaid optical cell has an LOQ of 693 nM for absorbance and an LOQ of 156 nM for fluorescence of rhodamine B, where the 0.1 µM sample was not readily detected, but the 0.25 µM sample is clearly discernible in Table 1 and Figure 5.

### 3.4. Precision

The results from Figure 5c are tabulated in Table 1, showing average optical data at each concentration along with the standard deviation of the triplicates. When applying the linear fit, the absorbance measurements had the lowest standard deviation of 2.3 nM on the 1 µM sample, and the highest standard deviation of 94 nM on the 5 µM sample. Similarly, the fluorescence measurements had the lowest standard deviation of 9 nM on the 2 µM sample and the highest standard deviation of 257 nM on the 10 µM sample. Ultimately both absorbance and fluorescence measurements had less than 3% relative standard deviation for concentrations higher than 1 µM. Commercially available spectrometers, such as the Turner Cyclops-7F, can typically attain 0.1% RSDs for samples less than 0.5 µM [48]. The absorbance reading of the lowest concentration could be improved by 5-fold by extending the optical path 5-fold from 20.2 mm to 100 mm, but this shifts the dynamic range [28]. The fluorescence signal-to-noise of the first sample was also poor. Collimating optics, such as closely coupled GRIN lenses, could improve the collection efficiency of the fluorescent light could be implemented to improve the magnitude of the received signal. Otherwise, a fluid reservoir/well with a larger cross-sectional area could be used at the expense of more fluid volume. Similarly, a lock-in-amplifier could be applied to significantly improve SNR [46,49]. Nevertheless, for rhodamine B samples down to 0.25 µM, the inlaid optical cell produced accurate and consistent absorbance and fluorescence measurements that were clearly distinguished from noise.

### 3.5. Attenuation Coefficient

**Figure 6:**
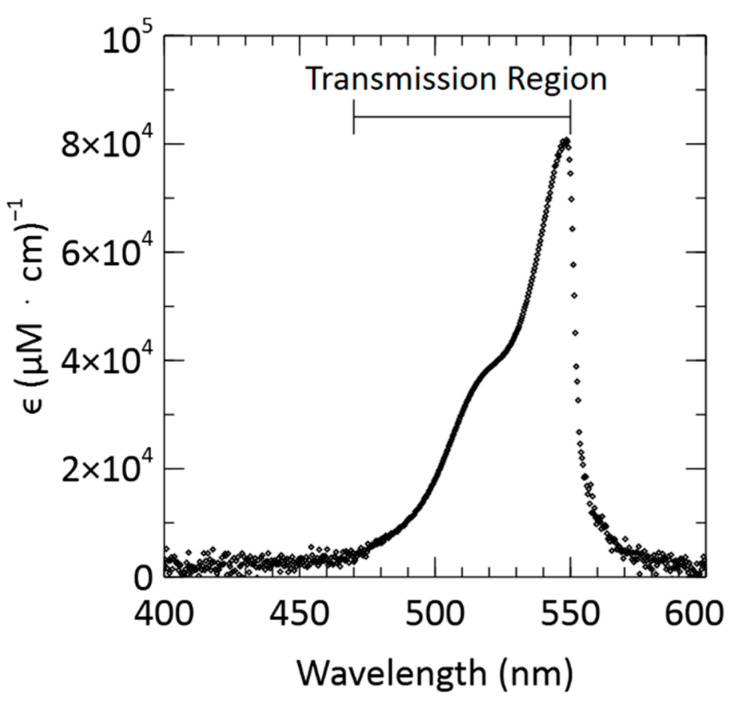
Attenuation coefficient, ϵ, of rhodamine B in Milli-Q water plotted against wavelength. The drop at wavelengths greater than ~550 nm is due to the cut-off of the excitation filter.

The attenuation coefficient, ϵ, of a species can be determined from the slope of its absorbance vs. concentration curve. The attenuation coefficient, ϵ, is a function of wavelength, λ, and is unique to the measured species:

(3) ϵ(λ)= M(λ)/l.

where the slope of the light absorbance calibration curve, M, is divided by the optical path length, l. M was determined at each wavelength by using the data shown in Figure 5a. Due to the use of the short-pass excitation filter, data obtained for wavelengths above ~550 nm are artificially near zero from the optical filter blocking those wavelengths. Similarly, the transmission spectra of the light source are limited to a 15 nm FWHM centred at 521 nm. The light source emits roughly 1% of its maximum at 470 nm. Consequently, the attenuation coefficient of rhodamine B was determined for a wavelength range of 470 nm to 550 nm—this is shown in Figure 6. Equation (3) was used to calculate each attenuation coefficient using the 20.2 mm optical path length of the fluid. The data in Figure 6 show a maximum attenuation coefficient of 80,774 (μM·cm)^−1^ at λ = 549 nm. As a comparison, the measured values of a range of rhodamine dyes by Yuan et al. showed the maximum molar extinction coefficient to be in the range of 80,000–120,000 (μM·cm)^−1^ [50]. Therefore, our measured values are like those reported in the literature. It is noteworthy, that the peak extinction coefficient may occur at slightly different wavelengths due to solvato-chromic effects. For example, a peak wavelength of λ = 545 nm has been reported for rhodamine B with methanol as the solvent [51], whereas we observe the peak in Milli-Q water at λ = 549 nm. Given that our samples are highly filtered, the attenuation coefficient and the molar extinction coefficients should be comparable as there are minimal scattering loses in the channel.

In summary, the novel optical cell presented here was thoroughly characterized and could be used in conjunction with numerous assays and a wide range of other lab-on-chip technologies. For example, embedded heaters as described in the work of Murphy et al. [44] could be integrated with this fluorescence chamber to realize an in situ RT-PCR chip. Similarly, the optical cell presented here can be readily integrated with on-chip valving solutions to control reagents and fluid flow for sample preparation, such as in the work of Luy et al. [27] or Ogilvie et al. [52], where reagents are combined with seawater samples for on-chip spectroscopy.

## 4. Conclusions

A microfluidic chip was successfully fabricated to validate a novel inlaid optical cell that simultaneously measures the light absorbance and fluorescence of injected fluids. This chip design uses embedded microprisms to couple light through the optical channel to excite contained fluids. The optical channel is shielded from external light sources by use of an inlaid opaque material. Two off-the shelf spectrometers were used to collect spectral measurements. Measurement consistency and sensitivity were evaluated through calibration testing by measuring the light absorbance and fluorescence of several prepared standards of rhodamine B over a concentration range of 0.1–10 μM and showed strong linearity. The limit of quantification for absorbance was found to be 693 nM and for fluorescence 156 nM. For measurements including and above 1 µM the relative standard deviation was below 3%. Our measured attenuation coefficient of 80,774 (μM·cm)^−1^ at λ = 549 nm was found to be within previously reported values. The inlaid microfluidic optical cell described and evaluated here will provide the foundation for many in situ sensors, for example, one that simultaneously uses the fluorescence and absorbance spectra of cellular pigments to identify harmful algal blooms in aquatic environments.

## Figures and Tables

**Table 1:** Average absorbance and fluorescence of measured rhodamine B samples with their associated standard deviations.

Conc. (µM)	Absorbance (AU)	Fluorescence (Counts)
0.1	0.0009 ± 0.0008	15 ± 7
0.25	0.0067 ± 0.003	54 ± 14
0.5	0.0250 ± 0.003	166 ± 14
1	0.0720 ± 0.0002	426 ± 8.4
2	0.1587 ± 0.004	963 ± 4.4
5	0.425 ± 0.008	2563 ± 45
10	0.8597 ± 0.004	4806 ± 125

## Data Availability

Not applicable.
